# Cold exposure impacts DNA methylation patterns in cattle sperm

**DOI:** 10.3389/fgene.2024.1346150

**Published:** 2024-02-20

**Authors:** Md Nazmul Hossain, Yao Gao, Michael J. Hatfield, Jeanene M. de Avila, Matthew C. McClure, Min Du

**Affiliations:** ^1^ Nutrigenomics and Growth Biology Laboratory, Department of Animal Sciences, Washington State University, Pullman, WA, United States; ^2^ Department of Livestock Production and Management, Faculty of Veterinary, Animal, and Biomedical Sciences, Sylhet Agricultural University, Sylhet, Bangladesh; ^3^ ABS Global, DeForest, WI, United States

**Keywords:** sperm, DNA methylation, epigenetics, imprinted genes, WGBS, embryonic development, osteogenesis

## Abstract

DNA methylation is influenced by various exogenous factors such as nutrition, temperature, toxicants, and stress. Bulls from the Pacific Northwest region of the United States and other northern areas are exposed to extreme cold temperatures during winter. However, the effects of cold exposure on the methylation patterns of bovine sperm remain unclear. To address, DNA methylation profiles of sperm collected during late spring and winter from the same bulls were analyzed using whole genome bisulfite sequencing (WGBS). Bismark (0.22.3) were used for mapping the WGBS reads and R Bioconductor package DSS was used for differential methylation analysis. Cold exposure induced 3,163 differentially methylated cytosines (DMCs) with methylation difference ≥10% and a *q-value <* 0.05. We identified 438 differentially methylated regions (DMRs) with *q-value <* 0.05, which overlapped with 186 unique genes. We also identified eight unique differentially methylated genes (DMGs) (*Pax6, Macf1, Mest, Ubqln1, Smg9, Ctnnb1, Lsm4,* and *Peg10*) involved in embryonic development, and nine unique DMGs (*Prmt6, Nipal1, C21h15orf40, Slc37a3, Fam210a, Raly, Rgs3, Lmbr1,* and *Gan)* involved in osteogenesis. *Peg10* and *Mest*, two paternally expressed imprinted genes, exhibited >50% higher methylation. The differential methylation patterns of six distinct DMRs: *Peg10, Smg9 and Mest related to embryonic development* and *Lmbr1, C21h15orf40* and *Prtm6 related to osteogenesis,* were assessed by methylation-specific PCR (MS-PCR), which confirmed the existence of variable methylation patterns in those locations across the two seasons. In summary, cold exposure induces differential DNA methylation patterns in genes that appear to affect embryonic development and osteogenesis in the offspring. Our findings suggest the importance of replicating the results of the current study with a larger sample size and exploring the potential of these changes in affecting offspring development.

## 1 Introduction

Epigenetic modifications like DNA methylation, histone modifications, and chromatin remodeling are heritable changes that can alter the transcription or affect the binding of transcription factors without altering the actual DNA sequences (Chamani and Keefe, 2019). In higher eukaryotes, the variation of gene expression among different tissues and cells is regulated by various epigenetic modifications (Raj, 2018). DNA methylation is one of the most studied epigenetic regulatory mechanisms (Moore et al., 2013). DNA methylation patterns of germ cells are critically important because they may propagate to the offspring and affect embryonic, prenatal, and postnatal development (Stewart et al., 2016). Acquiring epigenomic markers in sperm, particularly DNA methylation, occurs during the early embryonic development when primordial germ cells differentiate into male germ cells (GCs) and is completed during puberty (Stiavnicka et al., 2022). Male GCs undergo global erasure of DNA methylation through passive and active processes and become devoid of methylation after differentiation into pro-spermatogonia or gonocytes at 70–80 days post coitum in cattle (Costes et al., 2022). The gradual re-establishment of methylation then begins *via de novo* methylation and is maintained throughout adulthood across various phases of spermatogenesis (Seisenberger et al., 2013). After fertilization, the DNA methylation is erased in the paternal genome, except imprinted and a few other genomic regions which evade the epigenetic reprogramming and maintain the inherited methylation pattern in progeny (Elhamamsy et al., 2017). Imprinted genes exist in clusters, which is controlled by DNA methylation status of imprinting control region (ICR), resulting in the mono-allelic expression dependent on their parental origins (Sasaki and Matsui, 2008; Hudson et al., 2010). Sperm shows distinct global methylation patterns compared to somatic cells (Oakes et al., 2007; Cui et al., 2016). DNA methylation provide additional compactness to sperm nuclei and protect the DNAs from damage during the passage through female reproductive tract (Gannon et al., 2014). The entire differentiation, demethylation, remethylation and maturation processes of sperm are maintained in a sequential order. Proper establishment of methylation and maintenance of imprinted regions is required for optimal sperm motility, functionality and fertilization capability (Rotondo et al., 2021).

In mouse studies, exposure to a wide variety of environmental factors, such as cold or heat, toxicants, stress, and nutritional factors, alters DNA methylation in the germline, including sperm, resulting in “Epigenetic Memory” of parental exposures to environmental stresses (Martin and Fry, 2018; Nilsson et al., 2018; Raj, 2018; Skinner, 2018; Sun et al., 2018). Impaired sperm DNA methylation caused by environmental factors can affect post-fertilization epigenetic reprogramming, totipotency establishment, embryo development, and long-term phenotypic abnormalities in offspring (Wasson et al., 2013; Tang et al., 2016; Zhao et al., 2022). Loss of imprinting due to hypomethylation in silenced alleles may lead to overexpression of corresponding genes, which may reduce the fertilization capacity of sperm and elicit aberrant phenotypes in offspring (Elhamamsy et al., 2017).

The extensive grassland in the Pacific Northwest’s (PNW) valleys, hills, and plain areas has cultivated a vibrant beef cattle industry (William and Anderson, 1971). The temperature in the Northwest and most northern areas of United States and other regions around the world, varies profoundly between summer and winter seasons. Summer in PNW is generally moderate to mild, with an average temperature of 19°C (Vasquez, 2022). On the other hand, winter in PNW comes with rain, snow, ice and chilling temperature of an average between −3 and −6°C, which may go below −6°C.Like other homeothermic animals, production performance of cattle optimizes in their thermoneutral zone (TNZ) which ranges from −5 to 25°C (Avendaño-Reyes, 2012). Between this range cattle can effectively maintain their physiological body temperature while temperature below or above TNZ have a negative impact on performance (Kerr, 2016). From approximately December through March, beef cattle are exposed to severe cold (some areas with temperature below −17°C). In mouse studies, low temperature exposure of males had considerable influence on epigenetic modifications of sperm, which affected offspring development (Skinner, 2018; Sun et al., 2018). However, despite the known temperature variations, the effect of cold exposure on DNA methylation in cattle sperm has not been examined.

In the current study, we used the whole genome bisulfite sequencing (WGBS) to assess the methylation status of sperm collected from pure blood Angus bulls during the winter and summer, analyzed differentially methylated genes and discussed their probable impacts in biological processes and embryonic development.

## 2 Methods

### 2.1 Semen collection and preservation

A total of 5 pure blood Angus bulls were selected for semen collection. Semen samples were collected twice from each bull during early March 2019 designated as Winter samples and early June 2019 designated as Late Spring samples (Supplementary Table S3). Semen was collected from 5 pure blood Angus bulls by ABS Global, DeForest, WI (Supplementary Table S3). Because bovine sperm formation requires 61 days (Staub and Johnson, 2018), bulls were exposed to the coldest temperature (January and February) during spermatogenesis for semen collected in March (Winter group), and warm temperature (April and May) for semen collected in the early June (Late Spring group). During the winter of 2019, the average temperature during January to March was 0.5°C (max 6 to min -5°C) and during late spring the average daily temperature during the April and May were 8.01°C (max. 21 to min. 10°C) and 13.2°C (max. 28 to min. 18°C) respectively. All the semen straws were transported to the lab in a liquid nitrogen shipper and stored at −80°C temperature.

### 2.2 Isolation of genomic DNA

Semen samples from the same 3 randomly selected bulls out of 5 bulls collected during the winter and late spring respectively were used for isolation of genomic DNA as previously described (Perrier et al., 2018). One straw from each bull was used for DNA extraction (about 20 million spermatozoa). After thawing a straw at 37°C temperature, semen extender was removed by Phosphate Buffer Saline (PBS) wash and incubated with lysis buffer (10 mM Tris-HCl pH 7.5, 25 mM EDTA, 50 mM dithiothreitol, 75 mM NaCl, 1% SDS and 0.5 μg glycogen)) and proteinase K (0.2 mg/ml) at 55°C overnight. The phenol: chloroform (1:1) was used to extract the DNA after incubation with RNAse A for 1 h at 37°C and washed with ethanol. Extracted DNA quality was checked by using NanoDrop 2000 spectrophotometer (Nanodrop Technologies, USA).

### 2.3 Bisulfite conversion, library construction and WGBS sequencing

Bisulfite conversion and WGBS library were prepared by following a previously described method by Novogen (Zhou et al., 2018). High quality genomic DNA (2 µg) from each sample was used for library preparation and bisulfite conversion. Briefly, the genomic DNA spiked with lambda DNA were fragmented to 200–400 bp with a Covaris s220 sonicator (Covaris, Inc, USA) followed by terminal repair and adenylation. Lambda DNA was used as unmethylated control for bisulfite conversion rate calculation. Cytosine methylated barcodes were then ligated to the sonicated DNA and treated twice with an EZ DNA Methylation-Gold™ Kit (Zymo Research) for bisulfite conversion. Then, bisulfite converted libraries were sequenced on an Illumina HiSeq 2000/2,500 platform at the Novogene Bioinformatics Institute (Beijing, China).

### 2.4 Aligning and mapping bisulfite sequencing reads

FastQC was used to generate quality score of the sequences. Sequences with a PHRED score <20 and all adapter sequences were trimmed with Trim Galore program v 5.0 (Martin, 2011). The clean data from each sample were merged to align with the reference genome. The Cattle reference genome ARS-UCD 1.2 (GCA 002263795.2 https://ftp.ensembl.org/pub/release-108/gtf/bos_taurus/) incorporated with Y chromosome (GenBank: CM011803.1) were used for paired end alignment by indexing with bowtie2 (Version 2.4.5) under bismark (0.22.3, released: 19-11–2019) (Krueger and Andrews, 2011; Langmead and Salzberg, 2012).

### 2.5 Methylation calling and extraction

Bismark deduplication script “deduplicate_bismark” was used to remove reads in the same location from alignment output and the methylation information was extracted in CpG/CHG/CHH context by using “bismark_methylation_extractor” tool. To avoid the effect of bias towards non-methylation in the end of read due to end repairing, first 6 bp were ignored (Krueger and Andrews, 2011).

### 2.6 Identification of differentially methylated cytosines (DMCs)

Two popular Bioconductor packages Methylkit and DSS of R platform were used to perform the downstream analysis (Akalin et al., 2012; Feng and Wu, 2019). Sequence bases contained less than 10 uniquely mapped reads were filtered and removed across all the samples by Methylkit. Sequence bases containing at least 10 uniquely mapped read were termed as CpG10s and used for further analysis. Methylation percentage in each CpG site was calculated using the DMLtest function of DSS where a single CpG site containing greater than 10% difference and a *p*-value < 0.05 between the Late Spring and Winter groups were termed as a DMC. A DMC containing a higher mean methylation percentage during Winter in comparison with Late Spring group was termed as Hypermethylated DMC and similarly lower mean methylation percentage in Winter was term as hypomethylated DMC.

### 2.7 Identification of differentially methylated regions (DMR)

DMRs are genomic regions containing several adjacent DMCs and possessing a higher or lower methylation percentage in the experimental group. To form a DMRs, DSS first identifies statistically significant DMCs and merges several adjacent DMCs into a region (Feng and Wu, 2019). We calculated the mean methylation of CpG10s across all samples by using “dmlTest” function of DSS and generated the DMRs using default “callDMR” function with a *p-value < 0.05*. We also enabled the smoothing option of dmlTest function for besting estimation of mean methylation.

### 2.8 Annotation of differentially methylated regions

A popular R package Genomation operated on another R package GRanges was used for annotation of DMRs (Lawrence et al., 2013; Akalin et al., 2015). DMRs overlapped with different regions of the genes (promoters, introns, and exons) and distance from TSS were calculated by using “readTranscriptFeatures” and “getAssociationwithTSS” function of Genomation package. Promoters were defined as −2000 to −100 bp relative to the TSS sites. The association of DMRs with CGI was calculated by downloading CG data from California Santa Cruz Table Browser (Karolchik et al., 2004). During annotation with CGI, a CGI shore was defined as 4 kbp distance on the either side of each CGI. The list of annotated DMCs and DMRs is available in the Additional File 2: Supplementary Table S1 and Additional File 3: Supplementary Table S2.

### 2.9 Gene function analysis

All unique genes containing at least one DMR were further used for Gene function enrichment analysis by Database for Annotation, Visualization and Integrated Discovery (DAVID) (Huang da et al., 2009). Clusters of terms generated by DAVID showing EASE enrichment scores above 0.5 were considered significant. Along with this, Gene Ontology Enrichment analysis was performed by Gene Ontology Consortium’s online tool (http://www.geneontology.org/) to annotate the DMGs in Biological processes, Molecular functions and Cellular component categories (Mi et al., 2019). The identified unique genes were then classified and grouped into different pathways by using Reactome pathway and *p*-value <0.05 was considered as significant for each group (Fabregat et al., 2018).

### 2.10 Validation of DMRs by MS-PCR

To verify the DMRs, we performed MS-PCR of 6 genes *Smg9, Mest, Peg10*, *Lmbr1, C21H15orf40* and *Prmt6*. Genomic DNA was isolated from sperm samples with Proteinase K digestion. The quality of extracted DNA was checked using NanoDrop 2000 spectrophotometer (Nanodrop Technologies, USA) and samples with a A260/280 score above 1.8 were used for further analysis. Extracted DNA samples were used for Bisulfite conversion by EZ DNA Methylation-Direct™ Kit (Catalog No. D5020, Zymo Research, Irvine, CA, USA). Briefly, around 2 µg of the extracted DNA was mixed with 130 µL bisulfite conversion (BS) reagents and placed in a thermal cycler for bisulfite conversion with the following condition: 98°C for 8 min, 64°C for 3.5 h and stored 4°C until further use. Through BS conversion, unmethylated cytosines (C) were converted to uracil (U) while methylated cytosines remained unchanged. BS converted DNA samples were amplified by PCR with 1 sets of primers for methylated DNA and another set of primers for unmethylated DNA (Supplementary Table S4) designed by using MethPrimer website (https://www.urogene.org/methprimer/). The following PCR conditions were used for amplification: initial denaturation at 94°C for 5 min, followed by 40 cycles of 94°C for 30 s, annealing temperature of primers (53°C–58°C, varied among the primers) for 30 s and 72°C for 60 s; and final elongation for 7 min at 72°C (Ku et al., 2011). Amplified DNA products were electrophoresed for 25 min at 100V in a 2% Agarose gel. The gel was scanned, and the presence or absence of DMRs was determined based on the existence and strength of bands on the gel.

### 2.11 Statistical analysis and graphing

R package Methylkit, gplots, Dplyr, and Graph Pad Prism version 5.3.0 were used for PCA, Clustering, Heatmap generation and preparation of other figures. BAM files (*.bam) produced by Bismark were uploaded to SeqMonk software (http://www. bioinformatics. babraham.ac.uk/projects/seqmonk/) to visualize the location and methylation levels of genes. Data are reported as mean ± SEM. Two-tailed unpaired Student’s t-test was applied for the identification of significant difference (*p* < 0.05).

## 3 Results

### 3.1 Quality assessment of cattle sperm WGBS

**FIGURE 1:**
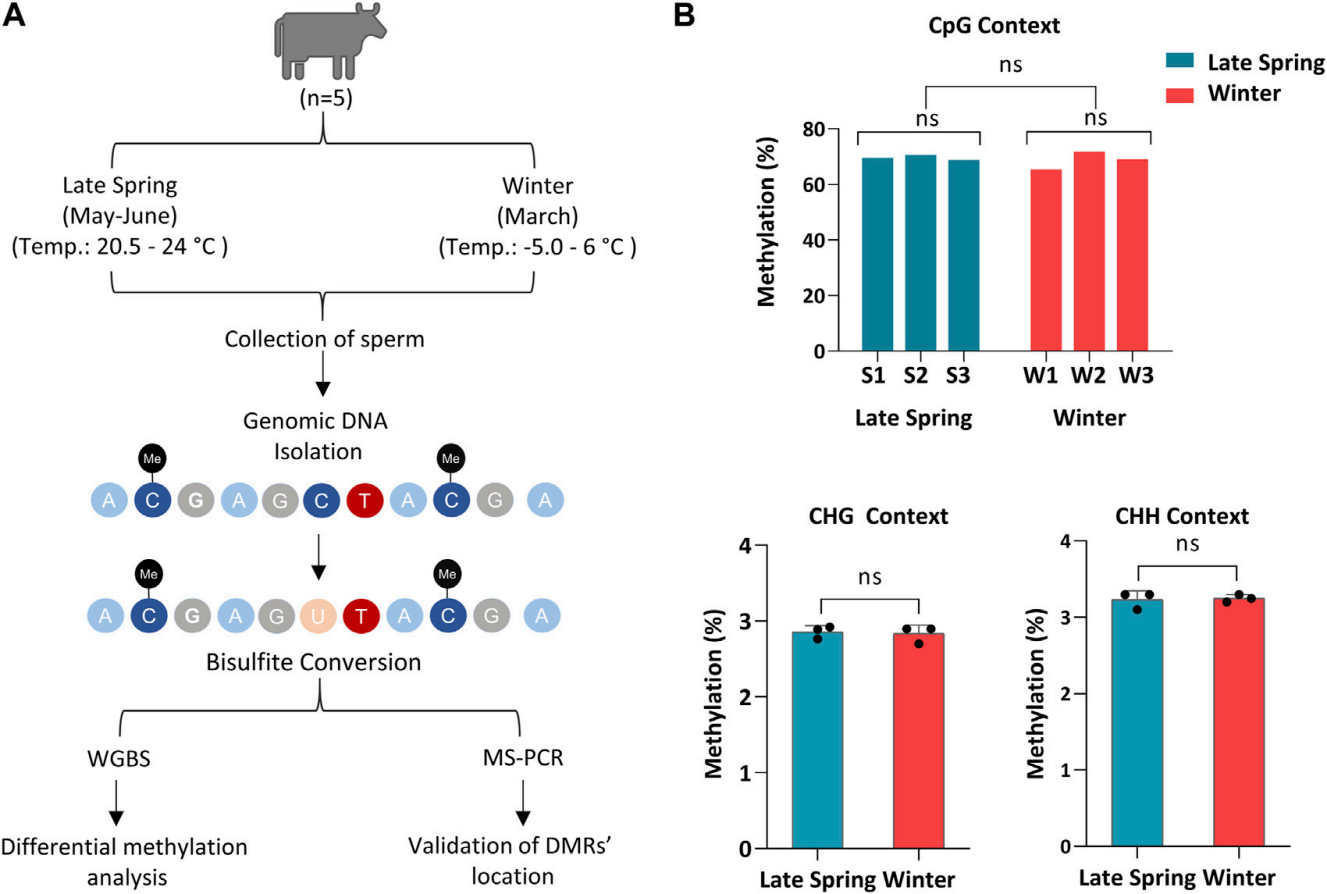
Whole Genome Bisulfite sequencing of cattle sperm during late spring and winter. **(A)** Schematic diagram of overall experimental design and procedures. **(B)** Methylation level of sperm on CpG, CHG and CHH (non-CpG) context. CHG and CHH: H corresponds to A, T or C. S1, S2, S3: Samples from Late Spring and W1, W2, W3: Samples from Winter.

Semen straw samples produced during the late spring and winter from 5 different bulls were collected by ABS Global, of which sperm from three bulls in late spring and winter, respectively, were randomly selected and sequenced using WGBS (Figure 1A). The sequence statistics and alignment statistics are summarized in Table 1. WGBS generated an average of 112.3 ± 3.41 million reads (Table 1) with an average Phred quality score of 36% which indicates an overall high sequence quality. The bisulfite conversion rate averaged 99.7% ± 0.02%. The sequence alignment was conducted using bovine reference genome ARS-UCD 2.1 incorporated with Y chromosome Data (GenBank: CM011803.1), which exhibited 79.5%–82.7% of uniquely mapped reads across all the samples (Table 1). An average of 55.9 million reads were mapped uniquely with an average of 70% reads aligned on CpGs. Additionally, we detected on average 2.83% ± 0.01% of CHG and 3.24% ± 0.02% of CHH were methylated in sperm samples (Figure 1B). Sequence and alignment statistics listed in Table 1 did not show statistical differences when comparisons were made between winter and late spring groups. In brief, high quality sequencing data on DNA methylation were obtained from all sperm samples, which facilitated the subsequent downstream analysis.

**TABLE 1:** An overview of basic statistics of sperm cell whole genome bisulfite sequencing (WGBS) and mapping quality using Bismark (Bowtie 2).

Parameters	Late spring			Winter		
	S1	S2	S3	W1	W2	W3
Sequencing statistics						
Number of Raw Reads (10^6^)	109.8	113.0	110.8	101.5	127.2	111.7
Average Phred Quality Score	35	35	36	36	36	36
Number of Sequence pair analyzed (10^6^)	54.5	56.3	55.1	50.5	63.5	55.7
**Alignment statistics**						
Number of uniquely aligned reads (10^6^)	43.4	45.8	44.2	41.9	51.1	44.6
Uniquely map reads (%)	79.57	81.26	80.01	82.75	80.44	80.03
Reads mapped to multiple loci (%)	14.35	12	12.50	10.24	12.51	12.87
Reads unmapped (%)	6.08	6.75	7.48	7.01	7.06	7.09
Bisulfite Conversion Rate (%)	99.75	99.71	99.7	99.69	99.7	99.69

Libraries were prepared from sperm samples DNA, collected from the 3 bulls during the late spring and winter seasons. Raw reads were obtained for trimming adapters and low-quality sequences. Uniquely mapped reads show a percentage of uniquely mapped reads with reference genomes (ARS-UCD, 1.2 incorporated with Y chromosome GenBank: CM011803.1). The bisulfite conversion rate denotes the percentage of C converted to uracil during Bisulfite conversion. S: late spring, W: winter.

### 3.2 Comparison of genome-wide CpG methylation between two seasons

**FIGURE 2:**
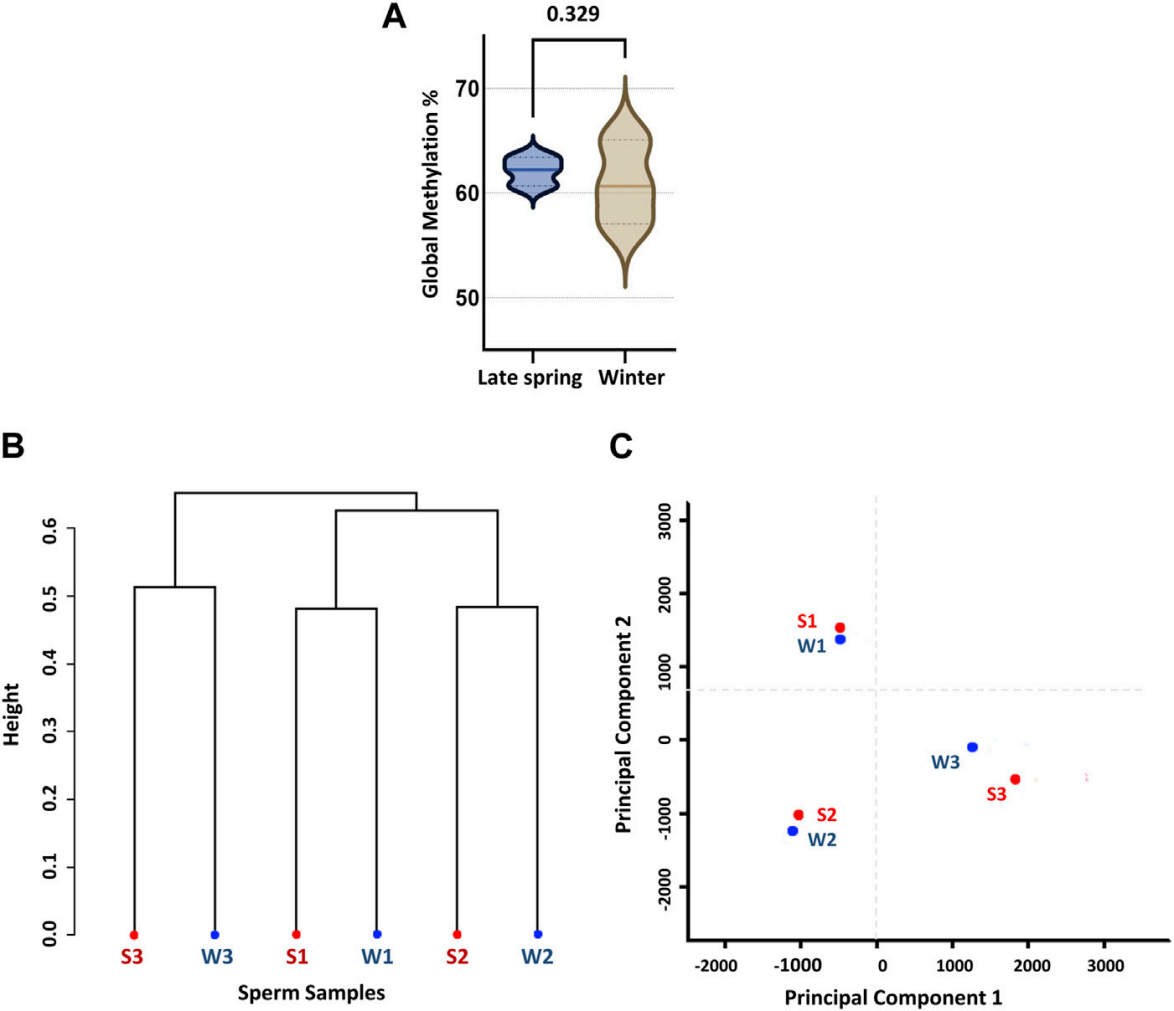
Global CpG methylation percentage and unsupervised clustering of sperm samples according to the season. **(A)** Global methylation percentage during late spring and winter. **(B)** Dendrogram clustering of methylation level of six sperm samples collected during late spring and winter. **(C)** Principal component analysis (PCA). S: Late Spring and W: Winter. Red dots represent samples during late spring and blue dots represent samples during winter.

For further analysis, we solely focused on methylation data on CpG context in order to obtain an overall scenario of genome-wide CpG methylation. In accordance with prior WGBS investigations of cow sperm samples, we found a genome-wide CpG methylation rate of 62.1% ± 2.3% and 60.7% ± 0.79% during the late spring and winter, respectively (Zhou et al., 2018), with no difference (*p* = 0.329) across two seasons (Figure 2A). To confirm, we performed an unsupervised hierarchical clustering analysis (Figure 2B) and principal component analysis (PCA) (Figure 2C). Clustering could not segregate samples according to the treatment groups and methylation difference among the individual samples was higher than the difference between groups. Additionally, PCA did not segregate the samples into two different groups, demonstrating that seasons had no impact on the global DNA methylation levels of sperm.

### 3.3 Identification of DMCs between two seasons

The CpGs covering at least ten uniquely mapped reads were filtered for further analysis by MethylKit and named CpG10 (Akalin et al., 2012). Around 30% of CpGs fell under the category of CpG10 and had an average methylation percentage of 26.3%. Among CpG10s, 33% had a methylation level <20% (hypomethylated), more than 60% had methylation level within 20%–80% (intermediate) and around 10% had methylation level more than 80% (hypermethylated) (Table 2). But the number of hypomethylated, intermediate and hypermethylated CpG10s did not differ significantly between late spring and winter groups.

**TABLE 2:** Comparison between overall sperm DNA methylation during late spring and winter.

Parameters	Late spring (n = 3)	Winter (n = 3)
Total number of CpG analyzed at CpG10 (10^6^)	16.74 ± 1.63	17.95 ± 1.64
Percentage of CpG10	29.1 ± 1.45	30.81 ± 1.52
Average DNA methylation of CpG10	26.3 ± 2.67	26.2 ± 2.79
Percentage of hypermethylated CpG10 (DNA methylation >80%)	10.33	7.45
Percentage of intermediate CpG10 (DNA methylation in 20%–80%)	55.82	64.70
Percentage of hypomethylated CpG10 (DNA methylation <20%)	35.67	29.85

Mean values were presented with Standard Error as Mean ± SEM. CpGs covered by at least 10 uniquely mapped reads were denoted by CpG10. A *p*< 0.05 were considered as significant, but there was no significant difference between late spring and Winter samples in the first 4 parameters. SEM: Standard error of the mean.

Next, we ran unsupervised hierarchical clustering (Supplementary Figure S1A) and PCA (Supplementary Figure S1B) of filtered CpG10s. Consistent with previous findings, PCA and clustering could not distinguish samples into two different groups. Taken together, cold exposure of bulls during winter did not induce changes in overall methylation of CpG10s and the effect of inter-individual variability was higher than the effect of cold exposure.

**FIGURE 3:**
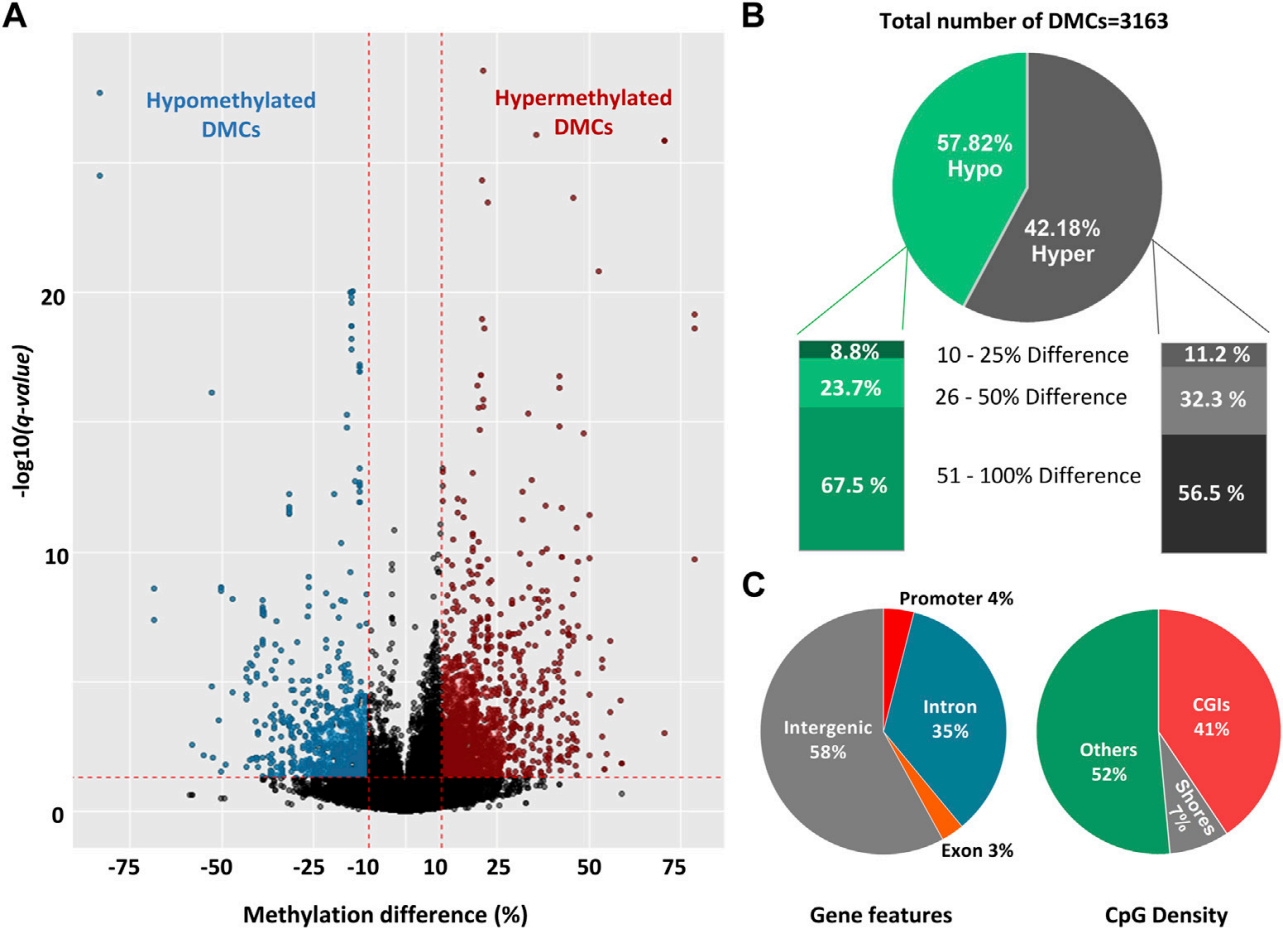
Differentially methylated cytosines (DMCs) in sperm collected during late spring and winter. **(A)** Volcano plot of DNA methylation (%) difference between sperm from late spring and winter. A total of 3,163 DMCs were detected with 10% difference and *q-value* < 0.05 are highlighted on both sides of the plot. DMCs highlighted with blue colors are hypomethylated and red colors are hypermethylated. **(B)** Percentage of hypomethylated and hypermethylated DMRs between two seasons. **(C)** The distribution of hypermethylated DMCs across different gene features includes exons, introns, promoters, intergenic regions, and CGI. CGI: CpG island, DMC: Differentially methylated cytosine, DMR: Differentially methylated region.

To reveal the effect of seasons on DNA methylation, we analyzed 92,622 CpG10s obtained by filtering across 6 sperm samples. We applied a moderate threshold of DNA methylation difference ≥10% and *p* < 0.05 and R Bioconductor package Methylkit was able to detect 22 DMCs. Furthermore, we obtained 3,163 DMCs with a threshold of methylation difference ≥10% and *q-value* < 0.05 (Figure 3A; Supplementary Table S1) by R Bioconductor package DSS (Feng and Wu, 2019). Among the identified DMCs by DSS, methylation difference ranged from 12% to 82% across two groups and around 90% of the DMCs had methylation difference between 25% and 100% (Figure 3A). Therefore, we used a stricter threshold of *q-value* < 0.001 and methylation difference ≥25%, and only 8% of the previously identified DMCs fallen into this category. In the rest of the study, we used the threshold of ≥10% difference and *q-value* < 0.05 for identification of DMCs. The ratio of the hypermethylated and hypomethylated DMCs were 57.8% and 42.2% respectively (Figure 3B). Distribution of DMCs also varied between hypomethylation and hypermethylation groups, with ∼63% DMCs showed more than 50% difference in methylation percentage and ∼27% DMCs had an over 25% difference in the percentage of methylation (Figure 3B).

All the DMCs were then annotated with R Bioconductor package “Genomation” relative to the gene and gene features (Figure 3C). The majority of DMCs (93%) were spread over intergenic regions and introns while only 4% overlapped with the promoters. For CpG features, around 52% of DMCs were annotated outside of the CpG Islands (CGIs) or CpG shores (4 kb on both sides of CGI) while around 41% of the DMCs overlapped with CGIs. Furthermore, DMCs were spread over all the chromosomes (Supplementary Figure S2)

### 3.4 Differentially methylated regions analysis

**FIGURE 4:**
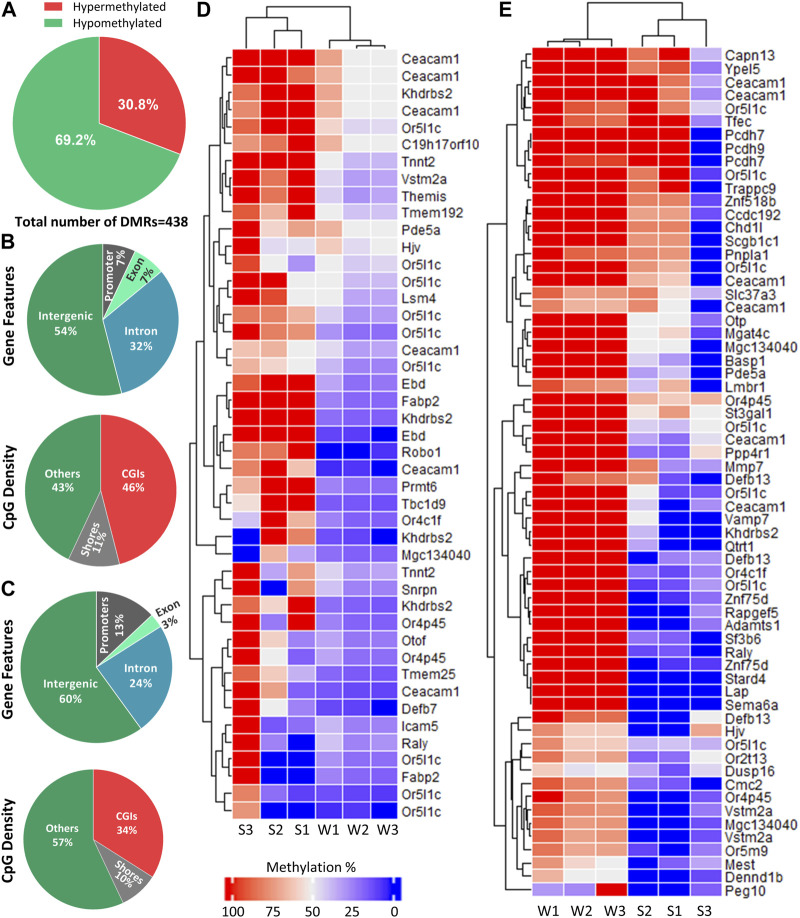
Distribution of DMRs across different genomic regions. **(A)** Percentage of hypomethylated and hypermethylated regions between late spring and winter. A total of 438 DMRs were detected among which 135 were hypermethylated and 303 were hypomethylated. **(B)** Distribution of hypermethylated DMRs across different gene features and CGIs. **(C)** Distribution of hypermethylated DMRs across different gene features and CGIs. **(D)** Heatmap showing methylation percentage of top 44 differentially hypomethylated DMRs have *q-value* < 0.05 on different DMGs. **(E)** Heatmap showing top 65 hypermethylated DMRs have a *q-value* < 0.05 on different DMGs. Each cell in the heatmap is colored according to the methylation level in each DMR. DMR: Differentially methylated region, CGI: CpG island; DMG: Differentially methylated gene, S: Late Spring, W: Winter.

Differential methylation analysis was performed by using DSS. We used default DMR calling function “callDMR” of DSS to identify significant DMRs with a *q-value* < 0.05. DSS identifies several significant DMCs located close to each other and merge them to form a DMR. DSS was able to detect 438 DMRs (Supplementary Table S2), and the majority of the DMRs were hypomethylated (69.2%) (Figure 4A). The DNA methylation percentage of hypomethylated and hypermethylated DMRs was able to cluster samples according to two seasons on the heatmap (Figures 4D, E).

DMRs were then annotated to identify their overlapping with introns, exons, promoters, intergenic regions, CGIs, and shores. Among the hypermethylated DMRs, 60% overlapped with intergenic region and 13% overlapped with promoters. Regarding regions overlapped with CpG density, 34% overlapped with CGIs (Figure 4B). The annotation of hypomethylated regions exhibited similar trends (Figure 4C). Most hypomethylated DMRs overlapped with intergenic regions while only 7% overlapped with promoter regions. In terms of CpG density, most DMRs overlapped with CGIs (46%) (Figure 4B).

**FIGURE 5:**
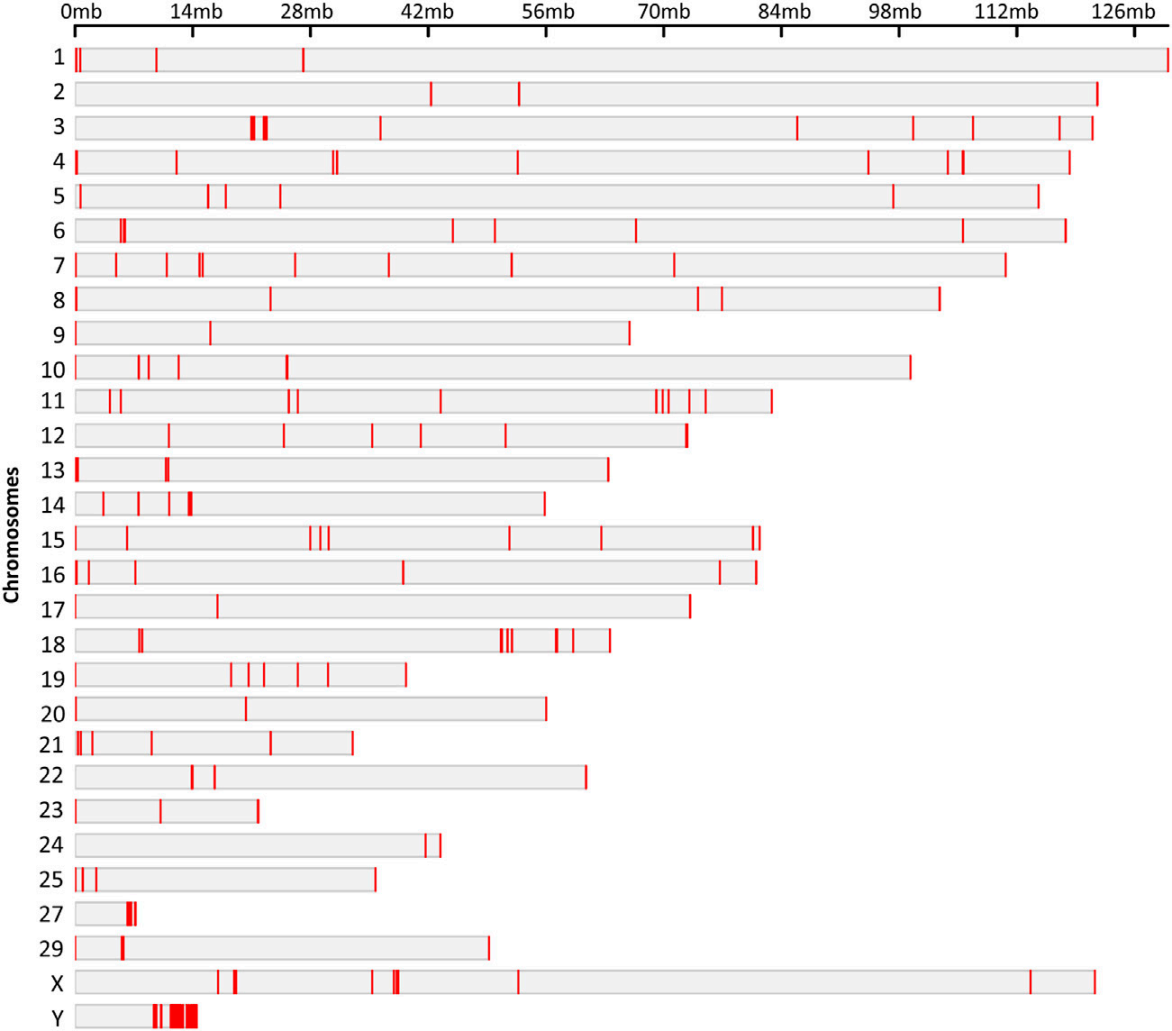
Chromosomal locations of the DMRs. The chromosome number and size are presented in reference to the *Bos Taurus* genome. The chromosomal location of each DMR is marked with red color on corresponding chromosome.

Hypo and hypermethylated DMRs were dispersed on almost every chromosome except chromosomes 26 and 28 which did not contain DMRs. Surprisingly, 50% of the hypomethylated DMRs were present on four chromosomes: 13, 16, 18 and Y, while Y chromosome contained 92 hypomethylated DMRs (Figure 5).

### 3.5 Functional enrichment and Gene Ontology analysis of the differentially methylated genes

**FIGURE 6:**
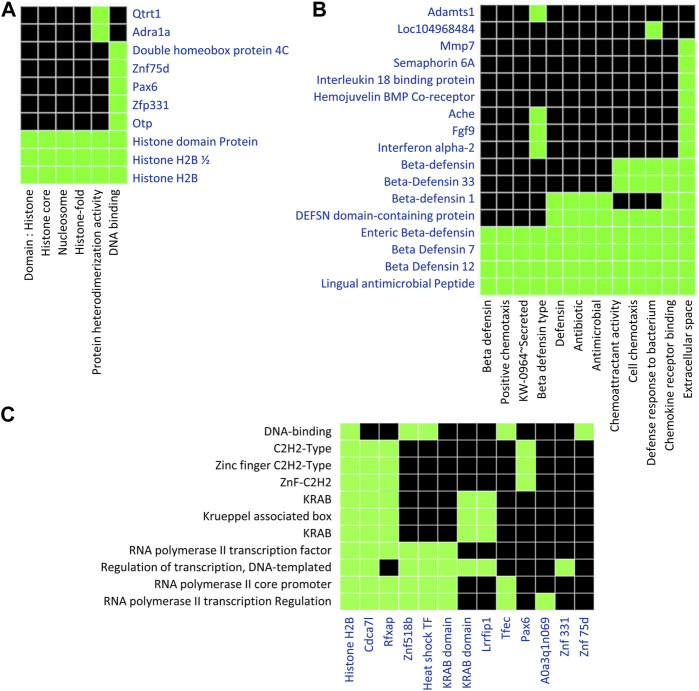
Functional Enrichment analysis using DAVID tools focused on genes containing DMRs. A list of 186 unique genes containing one or more DMRs was used for DAVID functional enrichment analysis. **(A)** The first diagram represents genes clustering across categories related to histone modification. **(B)** The second diagram represents a cluster of genes related to chemotaxis. **(C)** The third diagram represents a cluster of genes related to transcription regulation. Default settings of the DAVID bioinformatics tool were applied. Green represents the association of genes with particular Gene Ontology terms.

We performed the functional enrichment analysis in order to determine the biological processes and molecular functions of differentially methylated genes. We used Database for Annotation, Visualization and Integrated Discovery (DAVID) for functional enrichment analysis of 186 differentially methylated genes overlapped with 438 DMRs, among which 142 genes were distributed in 17 clusters among which top 3 clusters (Figures 6A–C) were associated with biological processes such as chemotaxis, transcription regulation from RNA polymerase II promoter, and histone modification.

**FIGURE 7:**
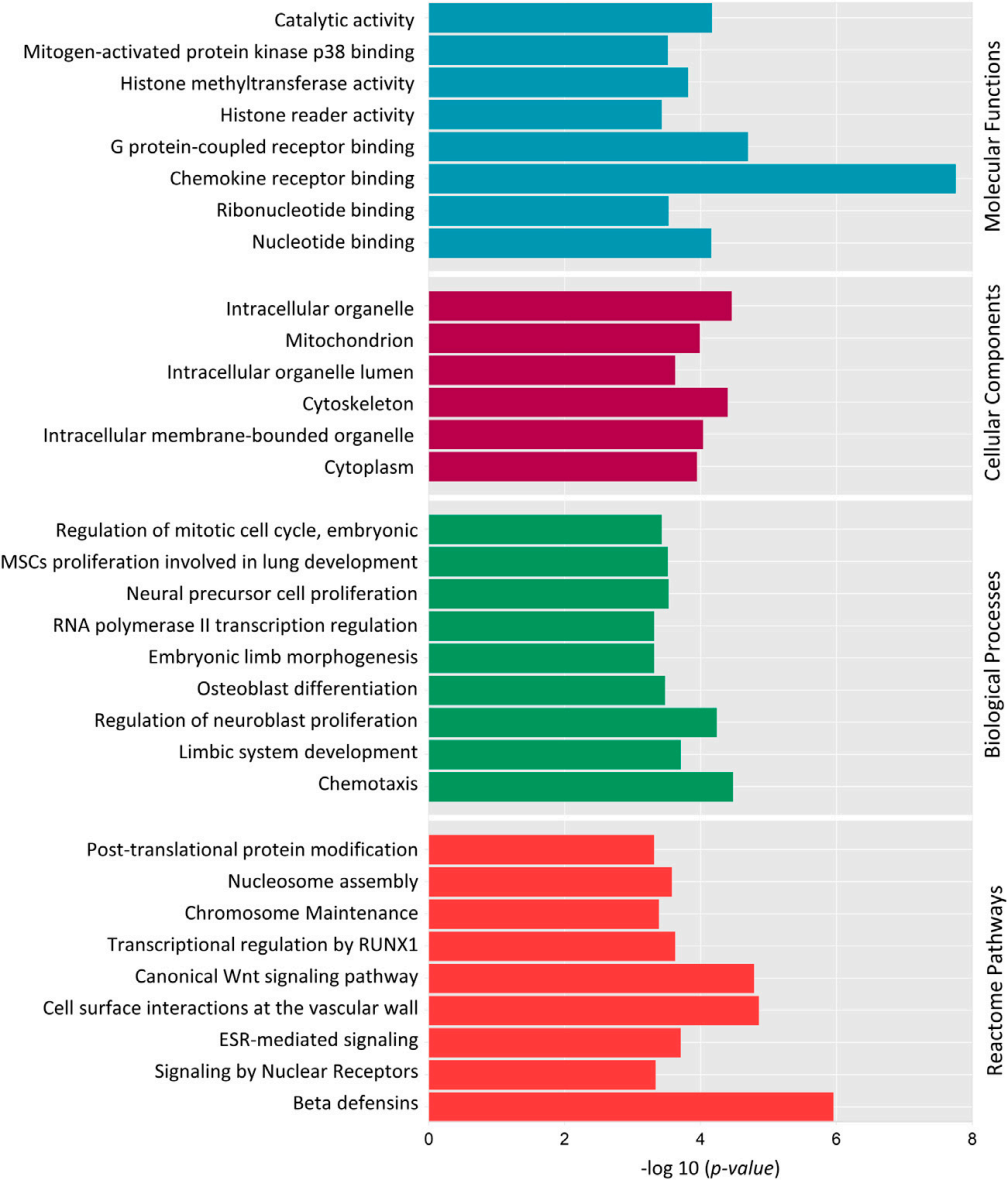
Gene Ontology (GO) and Reactome pathway enrichment analysis of 186 differentially methylated genes. Top significantly enriched GO terms associated with Molecular Functions**,** Cellular components and Biological Processes are listed. Reactome pathway enrichment analysis reveals the top pathways in which the DMGs are involved. *Y*-axis represents the GO term, and the *X*-axis represents the enrichment significance (-log10 (*p-value*)).

We also did a Gene Ontology (GO) Enrichment Analysis on 186 genes under the category of biological processes, cellular components, and molecular functions. Several GO terms identified by GO enrichment analysis matched those identified by DAVID. Several DMGs were associated in crucial biological processes: RNA polymerase II transcription regulation, osteoblast differentiation, regulation of neuroblast proliferation, limbic system development, regulation of embryonic mitotic cell cycle and chemotaxis (Figure 7). Along with this, DMGs were enriched in top molecular functions: chemokine receptor binding, histone methyltransferase activity, histone reader activity and G-protein-coupled receptor binding (Figure 7); and cellular component terms: mitochondria, intercellular organelle lumen, cytoskeleton, and cytoplasm (Figure 7).

Reactome pathway analysis was performed to determine the pathways in which the DMGs were involved (Figure 7). “Post-translational protein modification”, “Histone Methylation”, “Signaling by Nuclear Receptors”, “ESR-mediated signaling”, “Transcriptional regulation by RUNX1” and “Wnt Signaling pathway” were among top 20 identified significant pathways.

DAVID and GO enrichment analysis both demonstrated that, a large portion of DMGs are involved in common GO terms: chemotaxis, transcription regulation, histone modification and embryonic osteogenesis, neurogenesis and mitotic cell cycle. To summarize, the Reactome pathway analysis and GO enrichment analysis revealed that methylated genes might play crucial roles in embryonic neuroblast and osteoblast differentiation through the Wnt Signaling pathway, transcriptional regulation, and histone modification.

### 3.6 Differentially methylated genes related to embryonic development and osteogenesis

We performed a literature review of 186 differentially methylated genes (DMGs) and identified eight unique DMGs: *Pax6, Macf1, Mest, Ubqln1, Smg9, Ctnnb1, Lsm4,* and *Peg10* involved in embryonic development and nine unique DMGs: *Prmt6, Nipal1, C21h15orf40, Slc37a3, Fam210a, Raly, Rgs3, Lmbr1,* and *Gan* involved in osteogenesis in both animal and human studies (Table 3; Supplementary Figure S3).

**TABLE 3:** Differentially methylated genes (DMGs) related to embryonic development and osteogenesis across samples collected during winter and late spring.

Chr	DMR location			No. of DMCs	Differential methylation (%)	Methylation status	Gene name	Overlapping gene features			
	Start	End	Length					Promoter	Exon	Introns	CGI
**DMGs involved in early embryonic development**											
3	106792147	106792226	80	6	−54.401	Hypo	*Macf1*			Introns	CGI
8	76925715	76925810	96	10	−32.290	Hypo	*Ubqln1*	Promoter			CGI
18	51956744	51956850	107	9	−31.247	Hypo	*Smg9*	Promoter			CGI
22	13968071	13968223	153	7	−52.576	Hypo	*Ctnnb1*			Introns	CGI
4	94344895	94344962	68	5	54.463	Hyper	*Mest*			Introns	CGI
7	4893727	4893808	82	4	54.057	Hyper	*Lsm4*				CGI
15	62586466	62586665	200	12	31.940	Hyper	*Pax6*			Introns	CGI
4	12064511	12064698	188	9	31.104	Hyper	*Peg10*	Promoter		Introns	CGI
**DMGs involved in osteogenesis**											
3	3,6311879	3,6312012	134	9	−30.952	Hypo	*Prmt6*	Promoter	Exon		CGI
4	118276064	118276192	129	9	−55.807	Hypo	*Lmbr1*			Introns	CGI
4	103795994	103796062	69	4	−54.878	Hypo	*Slc37a3*	Promoter		Introns	CGI
6	66716994	66717074	81	9	−30.450	Hypo	*Nipal1*	Promoter	Exon	Introns	CGI
21	23263663	23263832	170	9	−31.060	Hypo	*C21h15orf40*	Promoter	Exon		CGI
8	102791262	102791354	93	7	47.265	Hyper	*Rgs3*			Introns	CGI
13	63412501	63412667	167	5	15.075	Hyper	*Raly*			Introns	CGI
18	7969785	7969847	63	4	51.942	Hyper	*Gan*			Introns	CGI
24	43445459	43445765	307	8	20.289	Hyper	*Fam210a*			Introns	CGI

Chr: Chromosome, DMR: differentially methylated region, DMC: differentially methylated cytosine, CGI: CpG island.

Among the genes involved in embryonic development, *Macf1, Ubqln1, Smg9, Ctnnb1* were hypomethylated and *Pax6, Mest, Lsm4,* and *Peg10* were hypermethylated. The identified DMRs overlapped with promoters of *Ubqln1, Smg9,* and *Peg10* and introns of rest genes (Table 3). Among the genes related to embryonic development, *Mes*t and *Peg10* are two paternally expressed imprinted genes that exhibited ∼50% increase in the methylation level.

Among the osteogenic genes, *Prmt6, Nipal1, C21h15orf40, Slc37a3,* and *Lmbr1* were hypomethylated in winter whereas others were hypermethylated. The identified DMRs had 30%–55% difference in methylation profile across two seasons and overlapped with promoter regions of *Prmt6, Nipal1, C21h15orf40,* and *Slc37a3;* in other genes, DMRs overlapped with the CGI of exons in seven genes and introns of three genes (Table 3).

### 3.7 Validation of DMRs by methylation specific PCR (MS-PCR)

**FIGURE 8:**
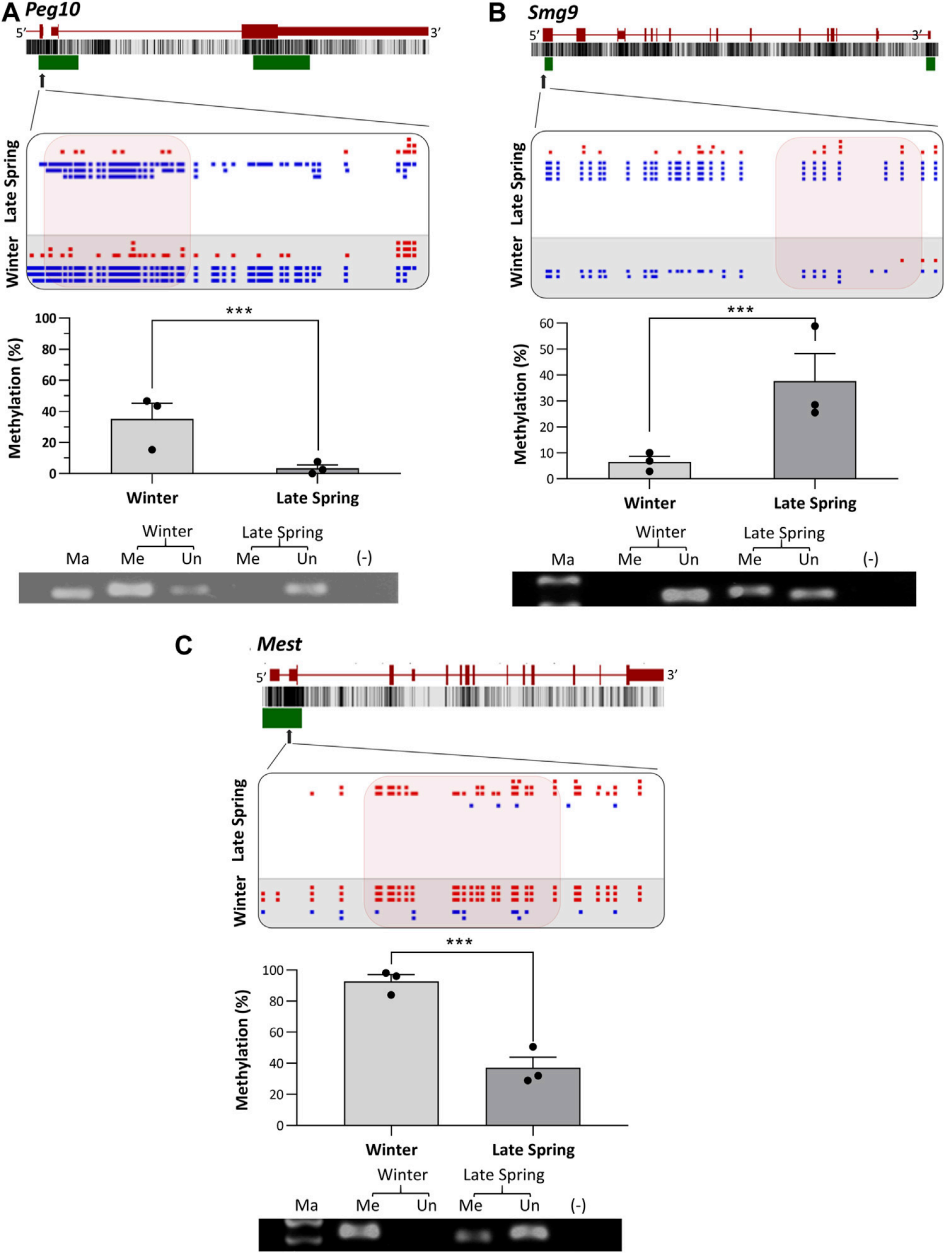
Cold induced methylation changes in 3 genes related to embryonic development during late spring and winter. **(A)** *Peg10*, **(B)** *Smg9* **(C)** *Mest.* For each gene, the genomic structure, CG percentage, CpG island, graphical display of DMRs from SeqMonk, methylation percentage calculated from WGBS and agarose gel image from MS-PCR are shown. The arrows point to the location of DMR in the actual gene structure and highlighted area shows methylation count of DMR on SeqMonk screenshot. In SeqMonk screenshot the red color dots on top represents methylated cytosine, and the blue dots on the bottom represents unmethylated cytosine. Each dot in red or blue color represents a unique read in each position. DMR: differentially methylated region, WGBS: Whole genome bisulfite sequencing, MS-PCR: Methylation-specific PCR, Ma: marker, Me: Methylated and Un: Unmethylated.

**FIGURE 9:**
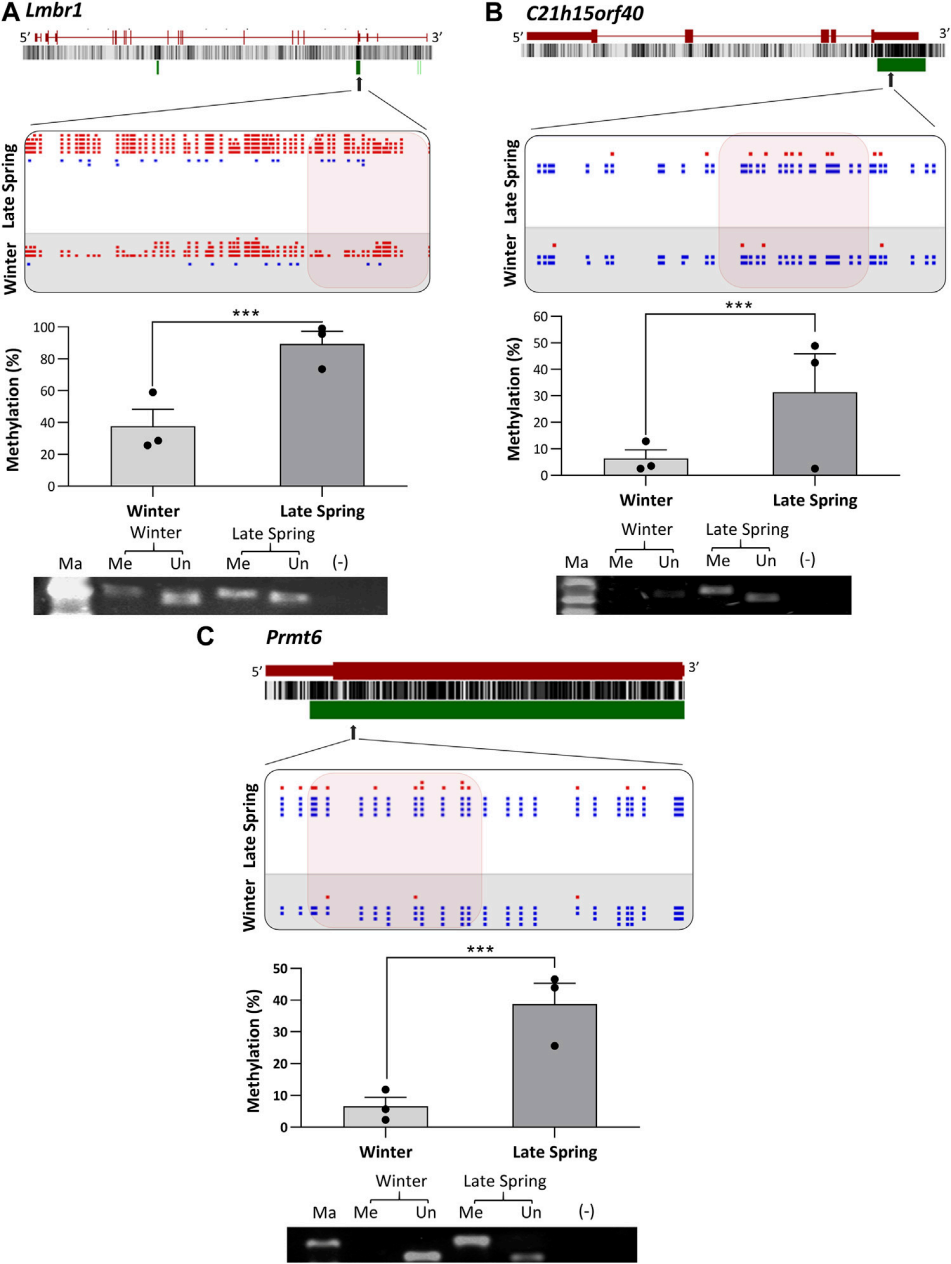
Cold induced methylation changes in 3 genes related to embryonic development during late spring and winter. **(A)** *Lmbr1*, **(B)** *C21h15orf40* **(C)** *Prmt6.* For each gene, the genomic structure, CG percentage, CpG island, graphical display of DMRs from SeqMonk, methylation percentage calculated from WGBS and agarose gel image from MS-PCR are shown. The arrows point to the location of DMR in the actual gene structure and highlighted area shows methylation count of DMR on SeqMonk screenshot. In SeqMonk screenshot the red color dots on top represent methylated cytosine, and the blue dots on the bottom represent unmethylated cytosine. Each dot in red or blue color represents a unique read in each position. DMR: differentially methylated region, WGBS: Whole genome bisulfite sequencing, MS-PCR: Methylation-specific PCR, Ma: marker, Me: Methylated and Un: Unmethylated.

To verify the location of the DMRs identified by WGBS, we performed MS-PCR of three randomly selected genes known to regulate embryonic development: *Smg9, Mest,* and *Peg10*, and three genes associated with osteogenesis: *Lmbr1, C21h15orf40,* and *Prmt6*. The bisulfite converted DNA was amplified with methylated and unmethylated primers designed by targeting specific regions of DMRs and electrophoresed in Agarose gel. Differential methylation quantified in WGBS and bands found from MS-PCR of the above-mentioned genes are compared in Figures 8, 9. In WGBS, *Peg10* and *Mest* had hypermethylated DMR with ∼30% higher methylation during winter which overlapped with their promoters’ region. In MS-PCR both genes showed stronger bands with winter samples amplified by methylated primers and strong bands in late spring samples with unmethylated primers (Figures 8A, C). On the other hand, *Smg9*, *Lmbr1, C21h15orf40* and *Prmt6* genes exhibited hypomethylated DMRs during winter. In MS-PCR all these genes had weak to no band during winter when amplified with methylated primers and stronger band during late spring amplified with unmethylated primers (Figure 8B; Figures 9A–C). The intensity of bands obtained from methylated and unmethylated primers corresponds with the results of WGBS and verifies the presence and absence of DMRS across two seasons. In conclusion, cold exposure induced differential methylation in genes associated with embryonic development, and osteogenesis and the identified DMRs overlapped with promoters and introns of genes including imprinted genes.

## 4 Discussion

The methylation profile of bovine sperm has recently come under the spotlight as a predictor of male fertility and a potential factor that might affect embryonic growth (Costes et al., 2022; Stiavnicka et al., 2022). Relation between sperm methylome and fertility has been revealed in human by comparing the methylation profiles between fertile and infertile male sperm (Laqqan et al., 2017; Oluwayiose et al., 2021). In human sperm, several external factors like smoking, alcohol, drug, obesity and stress can induce differential methylation of genes that activates post-fertilization and are critical for early embryonic development (Keyhan et al., 2021; Osadchuk et al., 2023). Similar to human studies, a comparison between sperm of fertile and subfertile bulls identified several DMRs that overlapped with genes involved in the capacitation of sperm, establishment of pregnancy, maintenance of chromatin structure, development of blastocysts, placental development and nervous system development, suggesting sperm methylation as a biomarker of bull fertility (Takeda et al., 2021; Tanaka et al., 2018; Capra et al., 2019; Luján et al., 2019; Costes et al., 2022; Llavanera et al., 2022; Stiavnicka et al., 2022).

Exposure to altered environmental variables like heat, cold, relative humidity, toxic chemicals including aflatoxin B1, air pollution, arsenic, bisphenol A, cadmium, chromium, lead, protein deficient diet, and high-fat diet, can impact methylation in germ cells during prenatal and post-natal life (Bind et al., 2014; Donkin and Barres, 2018; Kadayific et al., 2018; Martin and Fry, 2018; Skinner, 2018). Cold exposure or exposure to high ambient temperatures along with high relative humidity can also negatively affect the global or locus-specific DNA methylation independently or synergistically to produce a stronger impact (Bind et al., 2014; Toraño et al., 2016). However, no studies have reported the effects of bull cold exposure on the methylome profile of sperm or the subsequent implications on sperm fertility and embryonic development. In this study, we examined the impact of cold exposure on DNA methylation of cattle sperm and found a methylation level of approximately 75% across all CpG loci in the genome (Feng and Wu, 2019). Cold exposure did not induce a large-scale change in global methylation, rather the changes were localized in certain specific regions. We observed that the variability of methylation levels among individual cattle was quite large, which could originate from the variability of the sperm samples due to quality variations among different ejaculations (Jenkins et al., 2018).

Using Methylkit, with a threshold of methylation difference ≥10% and *p* < 0.05, used by another study for evaluating cattle sperm methylation, we could not detect DMCs, but with DSS, we got a striking difference (Stiavnicka et al., 2022). Consistently, DSS identified more DMCs (Piao et al., 2021). DSS quantifies methylation by the Bayesian hierarchical model based on direct methylation count, in contrast Methylkit uses logistic regression by converting the methylation count to percentage (Akalin et al., 2012; Feng et al., 2014). The difference in algorithm and counting method could be a possible reason for their difference in methylation quantification. Along with this, the algorithm of MethylKit is more biased toward genetic variations other than epigenetic variations during differential methylation analysis (Costes et al., 2022). Since we had a 10x sequencing depth and all bulls were pure blood Angus with low genetic variation, DSS outperformed Methylkit in terms of identifying DMCs as well as DMRs.

We identified around 438 DMRs spread over 189 genes by DSS. GO enrichment analysis of differentially methylated genes revealed their association with major biological processes: limbic system development, neuroblast proliferation, neural precursor cell proliferation, osteoblast differentiation, embryonic limb morphogenesis and regulation of embryonic mitotic cell cycle. Besides, Reactome pathway analysis reveals involvement of DMGs with canonical Wnt-β-catenin signaling, transcriptional regulation by RNA polymerase II promoter and post transcriptional protein modification. Wnt-β-catenin signaling is a major pathway that regulates embryonic axis formation, nervous system development and formation of vital organs throughout embryonic development (Tepekoy et al., 2015). Wnt-β-catenin signaling has a crucial role in implantation of blastocysts and embryo development in mammals (Xie et al., 2008). Altogether, both functional enrichment and pathway analysis highlighted the possible changes in embryonic development due to bull cold exposure.

Next, we identified DMGs involved in embryonic development and osteogenesis. We found eight unique DMGs: *Peg10, Mest, Pax6, Macf1, Ubqln1, Smg9, Ctnnb1* and *Lsm4* involve in embryonic development. *Peg10* is a paternally expressed imprinted gene derived from a retrotransposon and located in the *Sgce/Peg10* gene cluster on chromosome 4 in cattle (Lux et al., 2010). *Peg10* is highly expressed in placenta and promotes nutrient transfer from the mother to her fetus (Ono et al., 2006). Several mouse studies reveal that silencing or knockdown of *Peg10* causes embryonic lethality, impaired placental growth and trophoblast proliferation (Chen et al., 2015; Voon and Gibbons, 2016). Therefore, hypermethylation in the promoter region of *Peg10* may repress its expression and alter embryonic development (Ahn et al., 2020). Similarly, *Mest/Peg1*, hypermethylated in our study, is the first imprinted gene identified in mice and expressed in extraembryonic tissue at E6.5, and in mesoderm at E8.5 of mouse embryo (Kaneko-Ishino et al., 1995). Hypermethylation in the promoter region downregulated its expression and impacted the invasion of the extravillous trophoblasts with an impairment of embryonic growth (Rezvani et al., 2012). Also, increased methylation of *Mest/Peg1* was associated with abortion and pregnancy loss in the third trimester (Vasconcelos et al., 2019). *Pax6*, member of the *Pax* gene family, was first detected at E8.5 in neural progenitor cells of mouse developing forebrain, involving in retinogenesis and ocular tissue development (Meng et al., 2014). Knockdown of *Pax6* blocks neuroectoderm cell specification and offspring exhibit eye abnormalities (Zhang et al., 2010). Cytoskeletal crosslinking protein coding gene *Macf1* is a member of spectraplakin family and exhibits a lower methylation level after cold exposure in our current study. *Macf1* involves in Wnt/β-catenin signaling and plays a vital role in nervous system development during the embryonic stage (Cusseddu et al., 2021). C*tnnb*1, a major mediator of Wnt/β-catenin signaling pathway, hypermethylated in its promoter regions during winter (Tribulo et al., 2017). Hypermethylation of the *Ctnnb1* promoter inhibited the Wnt-signaling pathway and embryonic development (Gotze et al., 2010).

*Smg9* is a protein coding gene playing a critical role in nonsense-mediated mRNA decay and intellectual disability. Knockdown or impaired expression of *Smg9* is associated with heart and brain malformation and leads to abnormal embryogenesis (Shaheen et al., 2016; Rahikkala et al., 2022). *Ubqln1* is ubiquitin-like protein and involved in a wide variety of pathological and physiological processes, including body weight gain (Hiltunen et al., 2006). *Ubqln1* was hypomethylated after cold exposure. *Lsm4* is also known as Embryo Defective 1,644 or SM-Like protein 4, which was hypermethylated after cold exposure and lack of *LSM4* protein exhibited lethality to peri-implanted embryo in mice (Hirsch et al., 2000). Altogether, a common feature of all these genes is regulating the embryonic organogenesis and development.

We identified another nine unique genes: *Prmt6, Lmbr1, Slc37a3, Nipal1, C21h15orf40, Fam210a, Raly, Rgs3* and *Gan,* involving in osteogenesis during embryonic and adult life. While *Prmt6, Lmbr1, Slc37a3, Nipal1,* and *C21h15orf40* were found hypomethylated, while *Fam210a, Raly, Rgs3,* and *Gan* were hypermethylated during winter. Protein coded by *Prmt6* (Protein Arginine Methyltransferase 6) activates the AKT signaling pathway to promote the osteogenesis of mesenchymal stem cells (MSCs) (Veland et al., 2017). In an *in-vitro* study, overexpression of *Prmt6* inhibited osteogenic differentiation of dental stem cells (Wang et al., 2022). *Slc37a3* (Solute Carrier Family 37 Member 3) is important for osteogenesis, cell growth and differentiation of osteogenic cells (Surface et al., 2020). *Lmbr1* (Limb Development Membrane Protein 1), another hypomethylated gene during winter, is involved in osteoblast differentiation and embryonic appendage morphogenesis (Clark et al., 2001). Expression of *Lmbr1* is largely controlled by DNA methylation and altered expression is associated with acheiropodia and congenital hand abnormalities preaxial polydactyly (Ianakiev et al., 2001; Kim et al., 2010). *C21h15orf40* (chromosome 21 C15orf40 homolog) is a comparatively less studied gene, and a recent study suggested its role in regulating osteoblast differentiation and bone formation by interacting with Wnt and BMP pathways (Mohammadi et al., 2020). *Raly*, an RNA binding protein, contributes to pre-mRNA splicing and development of embryos. In *in-vitro* study, the downregulation of *Raly* reduced the differentiation capability of bone-derived mesenchymal stem cells (BMSCs) and the expression of osteogenic marker genes (Lin and Pan, 2021). Gigaxonin gene (*Gan*) mutation is associated with giant axonal neuropathy, an autosomal recessive neurological disorder, but recent studies reported its association with bone and hair abnormalities in human studies (Landrieu and Baets, 2013). Gigaxonin is an inducer of Sonic Hedgehog (Shh) signaling, which regulates osteogenesis and bone formation. In the current study, *Gan* had an overlap with a DMR with ∼50% hypermethylation during winter which might impair Shh signaling as well as osteogenesis. *Nipal1* (NIPA-like domain containing 1), which encodes a transporter for magnesium influx, was hypomethylated due to cold exposure. Overexpression of this gene is associated with elevated insulin content and negatively impacts bone mineral density (Ruppert et al., 2018; Manialawy et al., 2020). NIPAL-1 disruption was involved in degenerative spine conditions in a genome-wide study in humans (Manialawy et al., 2020; Zhang et al., 2021). *Fam210a*, regulates both muscle and bone development, was hypermethylated after cold exposure. *Fam210a* global knockout in mice induced *in utero* death of the embryos and decreased bone mineral density and bone biomechanical strength (Tanaka et al., 2018). Consistently, several reports showed that cold exposure affects the DNA methylation of osteogenic genes and bone development (Robbins et al., 2018; Shao et al., 2023). Therefore, the presence of altered methylation levels in all these genes in sperm might potentially impact osteogenesis in offspring.

Imprinted genes work in a parent-of-origin manner, and their monoallelic expression is controlled by differential methylation in the ICRs (Bartolomei, 2009; Bajrami and Spiroski, 2016). The paternal pronucleus undergoes genome-wide demethylation after fertilization but the imprinted genes and ICRs escape (SanMiguel and Bartolomei, 2018). The majority of the paternally expressed imprinted genes play key functions in placental development (Voon and Gibbons, 2016). Since *Peg10* and *Mest* are imprinted, the cold-induced methylome changes in these genes likely maintain through the post-fertilization reprogramming, affecting embryonic and fetal development with possible long-term effects on the growth performance of offspring.

There is growing evidence that altered DNA epimutation established in sperm can be transmitted to the offspring and the resulted phenotypic changes can transmit to multiple subsequent generations through transgenerational inheritance (Nilsson et al., 2018). The epigenetic changes induced by direct exposure to environmental factors in germ cells of F1 generation induced epigenetic alteration during the germ cell development of embryos, inducing transgenerational effects (Beck et al., 2017). Besides the ICRs of imprinted genes, a large number of specific regions in sperm maintain their methylation patterns during the post-fertilization genome-wide epigenetic reprogramming and transfer germ cell-specific epimutation to offspring (Keyhan et al., 2021; Sirard, 2022). In rodents, paternal physiological and metabolic adaptation to environmental factors are linked to the metabolic disorders in offspring (Rosenwald et al., 2013; Ng et al., 2014). Cold exposure induced differential methylation in several clusters of co-regulated genes in mouse sperm and altered their expression in adipocytes of offspring, resulting in hyperactive white adipose tissue (Sun et al., 2018). Altogether, cold exposure induces differential methylation patterns in sperm that may transfer to the offspring and impact embryonic development by regulating the expression of associated genes.

The exploratory nature and relatively small sample size are two limiting factors of our study. Increasing sample numbers would enable us to identify more DMRs and their locations more precisely. This deficiency is partially mitigated by our 10x sequencing depth and used two different pipelines to identify DMRs. Overall, this is the first study on cold-induced epigenetic reprogramming of bovine sperm and our analysis indeed identified and verified the presence of DMRs in the targeted regions induced by bull cold exposure. Further studies are warranted to test the effects of bull cold exposure on embryonic development and offspring growth performance.

In conclusion, bull cold exposure during winter alters the methylation levels at certain genomic loci and genes that can regulate early embryonic development and osteogenesis. Along with other genes, the changes of methylation in the promoters of imprinted genes suggest that bull temperature exposure could affect DNA methylation and the genomic imprinting of subsequent embryos and offspring. Our data underscore the necessity to determine the environmental exposure of bulls on the economic traits of offspring.

## Data Availability

The datasets presented in this study can be found in online repositories. The names of the repository/repositories and accession number(s) can be found below: https://www.ncbi.nlm.nih.gov/geo/; GSE222863.
