# Supplementary material for: Cold exposure impacts DNA methylation patterns in cattle sperm
Source: Front Genet. 2024 Feb 20;15:1346150. doi: 10.3389/fgene.2024.1346150 (PMC10912962; doi:10.3389/fgene.2024.1346150)
Supplement: Supplementary file 3 [file DataSheet1.pdf]

Supplementary Material

Cold exposure impacts DNA methylation patterns in cattle sperm

Md Nazmul Hossain, Yao Gao, Michael J. Hatfield, Jeanene M. de Avila, Matthew C. McClure and Min Du*

***Correspondence:** Min Du, min.du@wsu.edu

1. Supplementary Tables

Supplementary Table S1

List of significant DMCs of sperm whole genome bisulfite sequencing

Supplementary Table S2

List of significant DMRs of sperm whole genome bisulfite sequencing

Supplementary Table S3

Temperature at the day of sperm collection in late spring and winter

Seasons	Bull Id	Semen Collection date	Max Temp. (°C)	Min Temp. (°C)	Sperm Quality		
					Motility	Progressive Motility	Viability
Winter	2043	3/12/2019	-6	-16	51.2±2.14	41.8±1.88	60.1±2.48
	2040	3/14/2019	12	0			
	2102	3/14/2019	12	0			
	1996	3/26/2019	-1	-6			
	2026	3/29/2019	3	-4			
Late spring	2043	6/24/2019	32	20	53.2±2.58	41.4±1.07	62.2±1.15
	2040	6/27/2019	25	13			
	2102	6/12/2019	24	14			
	2026	6/14/2019	36	18			
	1996	5/24/2019	18	5			
Level of Significance					NS	NS	NS

Supplementary Table S4

Primer sequences for MS-PCR

Gene Name	Types of Primer	Sequences
Prmt6	Methylated	CGTTTGTTTTTAGTGCGTGAC
		GACCGCAATACTTTACTCGACTA
	Unmethylated	GGAGTGTTTGTTTTTAGTGTGTGAT
		CCAACCACAATACTTTACTCAACTA
Lmbr1	Methylated	TTTTTTGGAGGGAGATATAGTACGT
		AAAAATAACCGTAACGATTTTACGA
	Unmethylated	TTTTTTGGAGGGAGATATAGTATGT
		AAAATAACCATAACAATTTTACAAA
C21h15orf40	Methylated	CGAGTTTTAGGAATTAATCGAAATC
		GTACCTCTCCCCGAACGATA
	Unmethylated	GAGTTTTAGGAATTAATTGAAATTG
		CATACCTCTCCCCAAACAATAA
Smg9	Methylated	GCGGGTTTTTTATTTAAGGC
		CTATCCTAAATTAACCGATACCGAT
	Unmethylated	GTGGGTTTTTTATTTAAGGTGG
		TATCCTAAATTAACCAATACCAAT
Mest	Methylated	GTTGTCGTAGAGGATAAGTTCGC
		GACGAATTCATAAAACGACCG
	Unmethylated	GTTGTTGTAGAGGATAAGTTTGTGGT
		CAACAAATTCATAAAACAACCACTC
Peg10	Methylated	TGCGGATAGATATGAGTTGTAGAC
		TTTCAATACAAAAAAAACGCC
	Unmethylated	TGGATAGATATGAGTTGTAGATGG
		AATTTCAATACAAAAAAAACACC

2. Supplementary Figures

Supplementary Figure S1

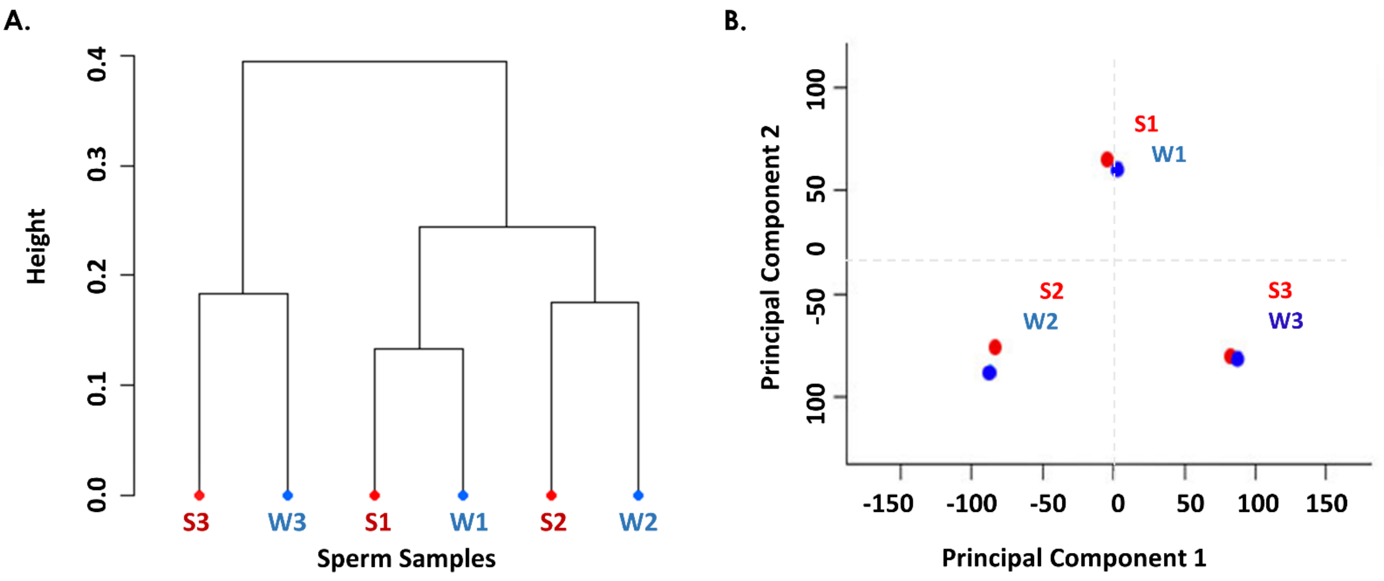

Supplementary Figure S1: CpG methylation profile of sperm during late spring and winter in CpG10. (A) Dendrogram clustering based on methylation level. **(B)** Principal component analysis (PCA) of CpG10. CpG10: CpGs containing at least 10 reads in each position across all samples.

Supplementary Figure S2

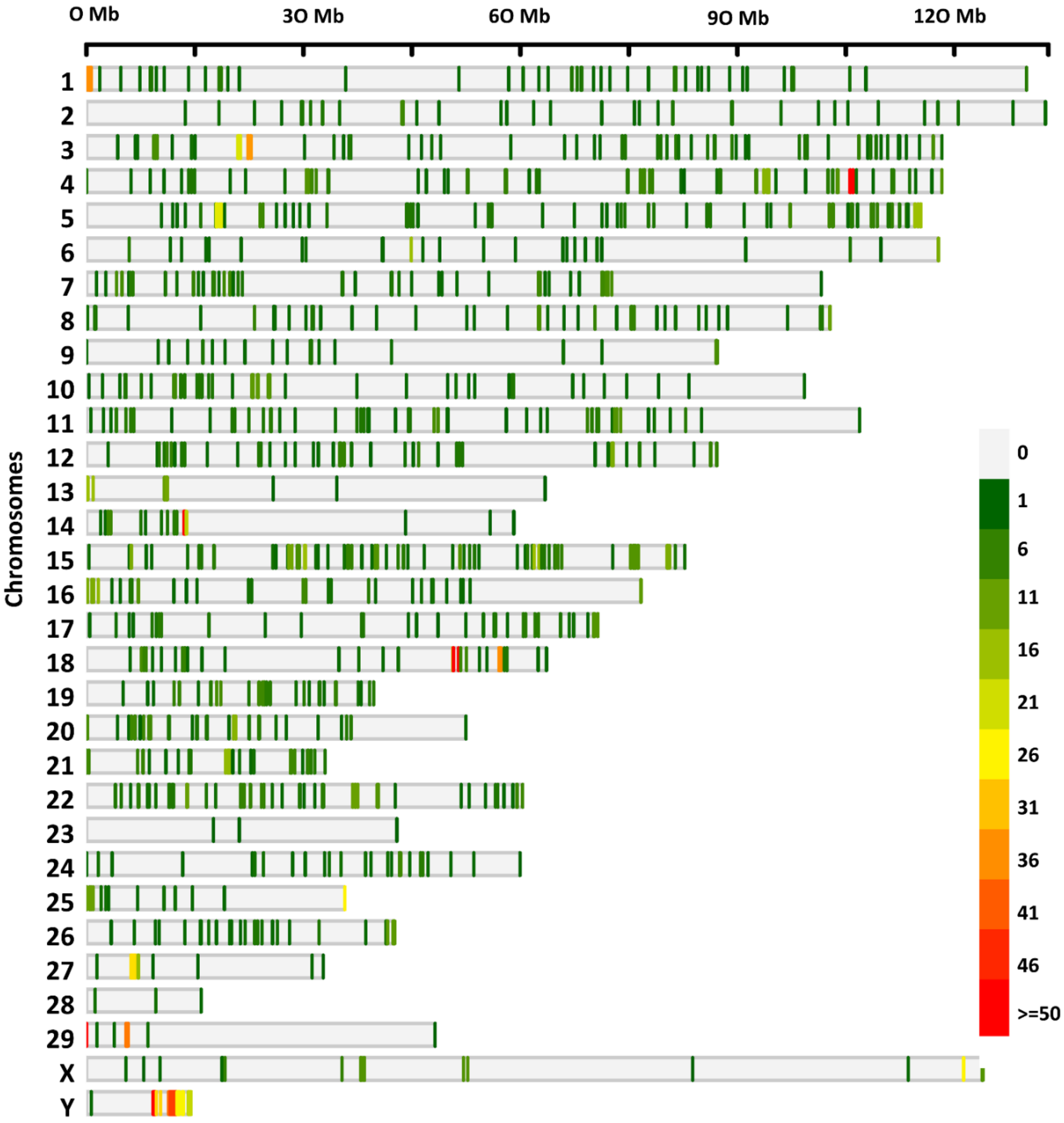

Supplementary Figure S2: **Chromosomal locations of the DMCs.** The chromosome number and size are presented in reference to the *Bos Taurus* genome. The chromosomal location of DMCs is marked with color on the basis of their number in different locations of chromosomes.

Supplementary Figure S3

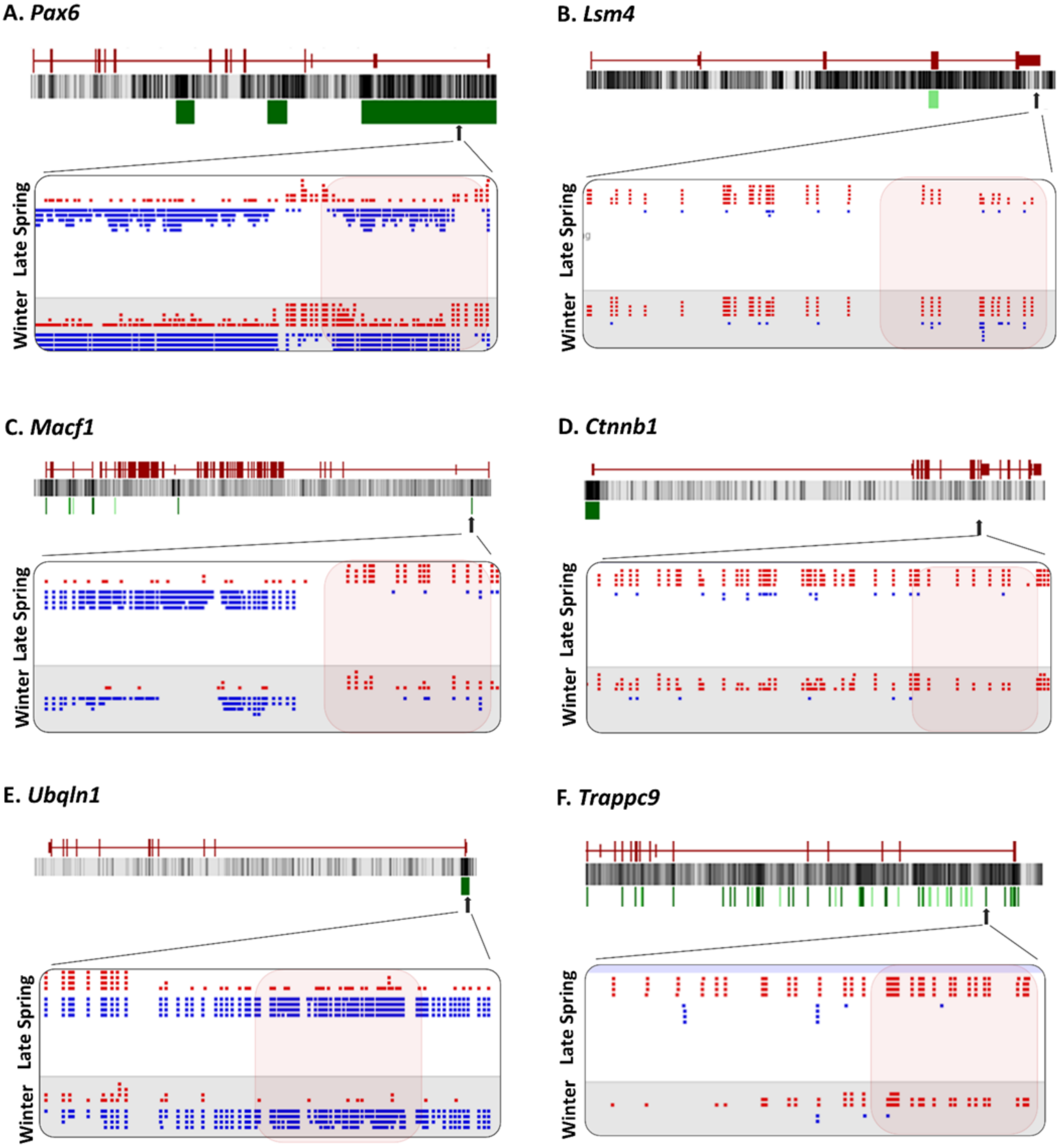

Supplementary Figure 3: **Cold induced methylation changes in 6 genes related to embryonic development during late spring and winter.** (A) *Pax6*, and (B) *Lsm4* were hypermethylated and (C) *Macf1*, (D) *Ctnnb1*, (E) *Ubqln1*, and (F) *Trappc9* were hypomethylated during winter. For each gene, the

genomic structure, CG percentage CPG island, graphical display of DMRs from SeqMonk are shown. The arrows point to the location of DMR in the actual gene structure and highlighted area shows methylation count of DMR on SeqMonk screenshot. In SeqMonk screenshot the red color dots on top represents methylated cytosine, and the blue dots on the bottom represents unmethylated cytosine. Each dot in red or blue color represents a unique read in each position. DMR: differentially methylated region.
